# IPSM-UNet: An Inverted Pyramid-Shaped U-Net++ Architecture with Multi-Resolution Information Interaction for Coronary Artery Segmentation

**DOI:** 10.3390/jimaging12050216

**Published:** 2026-05-20

**Authors:** Yinong Liao, Wei Li, Guopeng Liu, Rong Wang, Nan Zheng

**Affiliations:** 1 State Key Laboratory of Multimodal Artificial Intelligence, Institute of Automation, Chinese Academy of Sciences, Beijing 100190, China; liaoyinong2023@ia.ac.cn; 2 School of Artificial Intelligence, University of Chinese Academy of Sciences, Beijing 100049, China; 3 Chinese PLA General Hospital Medical School, Beijing 100853, China; liweiaffbb@163.com; 4 Department of Adult Cardiac Surgery, Senior Department of Cardiology, The Sixth Medical Center of PLA General Hospital, Beijing 100048, China; lgpll56@163.com (G.L.); wangrongd@126.com (R.W.)

**Keywords:** coronary artery segmentation, IPSM-UNet, U-Net++, feature aggregation, deep supervision

## Abstract

Accurate coronary artery segmentation is essential for diagnosis and interventional planning, but conventional U-shaped networks often miss thin, low-contrast vessels and break vessel continuity. We propose Inverted Pyramid-Shaped Multi-resolution U-Net (IPSM-UNet), a dual U-Net++ architecture with multi-resolution feature interaction, feature aggregation, and layer-wise deep supervision. The method is evaluated on DRIVE, CHASE_DB1, DCA1, and an internal coronary angiography dataset. IPSM-UNet achieves competitive or better performance across datasets, including F1 = 0.8310 and Acc = 0.9707 on DRIVE, Se = 0.8792 and Acc = 0.9745 on CHASE_DB1, F1 = 0.8043 and Acc = 0.9793 on DCA1, and Se = 0.8741, F1 = 0.8590, and Acc = 0.9879 on the internal dataset. IPSM-UNet improves vessel continuity and overall segmentation quality, particularly for small-caliber vessels, and supports downstream coronary analysis.

## 1. Introduction

Coronary arterial morphology is an important imaging cue for cardiovascular diagnosis, treatment planning, and follow-up assessment. In routine clinical practice, vessel delineation is still often performed or corrected manually by experienced clinicians, which is laborious, time-consuming, and sensitive to inter-observer variation. Automated coronary artery segmentation methods [1] therefore provide an important technical basis for quantitative vascular analysis. By extracting vessel morphology from medical images, these methods can help clinicians obtain structural information more efficiently [2] and support subsequent lesion assessment and interventional planning.

However, reliable coronary artery segmentation remains challenging. Coronary vessels have large anatomical variations in diameter, curvature, and branching patterns, and distal branches are often thin, low-contrast, and easily confused with background structures. In addition, X-ray imaging may be affected by motion artifacts, overlapping tissues, and noise, which further blur vessel boundaries. Traditional approaches [3] commonly depend on handcrafted descriptors [4,5], filtering strategies [6], or statistical modeling [7]. Although these methods can enhance certain vessel-like patterns, they usually have limited ability to represent complex vascular topology. Deep learning models, particularly U-Net-based architectures [8], have substantially improved segmentation performance, but missed tiny vessels and fragmented predictions are still frequently observed.

To address these limitations, we propose the *Inverted Pyramid-Shaped Multi-resolution U-Net (IPSM-UNet)*, a four-level U-Net-based segmentation framework. The working hypothesis of this study is that a U-Net-based model with stronger multi-resolution interaction and explicit shallow-layer supervision can improve segmentation performance, particularly for thin and low-contrast vessels, compared with conventional U-shaped networks. This hypothesis is motivated by the strong spatial-preservation capability of U-Net-like encoder–decoder architectures [8] and by the observation that vessel segmentation requires both fine boundary details and contextual continuity cues. IPSM-UNet therefore organizes two U-Net++-like branches into an inverted pyramid-shaped structure and promotes feature interaction across corresponding layers. By sharing intermediate features at selected central nodes and aggregating information from multiple spatial directions, the network improves the joint use of shallow details and deep contextual representations.

The main contributions of this work are summarized as follows:

- We develop IPSM-UNet for coronary artery segmentation, which strengthens multi-resolution information interaction and improves the representation of fine vascular structures.
- We design a feature aggregation module (FAM) that integrates features from adjacent sampling directions and different U-Net++ branches within the proposed spatial architecture.
- We introduce layer-wise deep supervision for the first-layer FAM outputs, enabling multi-level prediction fusion and improving the detection of small-caliber vessels while maintaining overall segmentation accuracy.

From a clinical translation perspective, the proposed approach aims to produce more continuous and complete segmentations of small, low-contrast coronary vessels in routine X-ray coronary angiography. Such vessel maps may provide clinicians with clearer structural cues for lesion assessment and pre-interventional planning, thereby supporting more consistent image interpretation.

## 2. Related Work

### 2.1. Vessel Segmentation Based on Neural Networks

Traditional segmentation methods generally partition an image into regions according to low-level cues such as intensity, color, texture, or shape. These hand-designed criteria are useful in controlled settings but are often insufficient for vessels with weak contrast and complex topology. Deep learning has changed this paradigm by learning task-specific representations directly from image data. Convolutional neural networks (CNNs) are able to extract hierarchical visual features without manual feature design. Hoffman et al. [9] introduced fully convolutional networks (FCNs) with skip connections for dense prediction, which provided an important foundation for later medical image segmentation models. Nevertheless, FCN-based methods may still suffer from limited boundary precision and overfitting when the training data are scarce.

Ronneberger et al. later proposed U-Net [8], whose encoder–decoder structure and skip connections made it highly influential in biomedical segmentation. Its compact design is easy to train, but the basic U-Net may not fully capture multi-scale vascular patterns. Many variants have therefore been developed. U-Net++ [10] introduces nested skip pathways to reduce the semantic gap between encoder and decoder features. MultiResUNet [11] replaces standard convolutional blocks with multi-kernel residual units to enlarge the effective receptive field. FR-UNet [12] further preserves full-resolution information through partial skip modules and cross connections. These designs improve segmentation quality, yet they may also introduce redundant connections or dispersed information flow, which can limit efficient feature aggregation for thin vessels.

Other architectures have explored different ways to enhance representation learning. Dense-UNet [13] uses dense connectivity to encourage feature reuse, and Dual U-Net [14] stacks two U-Net networks with an atrous spatial pyramid pooling (ASPP) module to incorporate contextual information. Attention-based models such as CS-Net [15] model spatial and channel dependencies to combine local and global cues. Despite their effectiveness, attention modules usually increase computational cost and may slow training, which motivates the design of convolution-based alternatives for efficient vessel segmentation.

In 2025 and 2026, vessel segmentation research further expanded beyond conventional static encoder–decoder designs. He et al. proposed TVS-Net [16], which incorporates temporal information from invasive coronary angiography through a 2D+time framework and a connectivity-preserving loss, showing that sequential information can improve vessel continuity in dynamic X-ray angiography. Kim et al. [17] demonstrated that self-supervised pretraining can improve the data efficiency and generalizability of coronary artery segmentation in coronary CT angiography, especially when annotated datasets are limited. Xu et al. introduced SADiff [18], which combines a dilated attention network, a diffusion-based subnet, and striped attention to suppress noise and refine tubular structures in coronary CTA. In X-ray coronary angiography, Ramos-Cortez et al. proposed a lightweight U-Net [19] based on structured kernel pruning to improve efficiency while maintaining competitive segmentation performance, which is attractive for resource-constrained clinical deployment. He et al. further proposed VM-CAGSeg [20], a vessel structure-aware state space model that integrates multiscale vessel priors with long-range dependency modeling and cross-stage feature fusion to enhance fine-grained coronary segmentation. For volumetric CCTA analysis, Khan and Liatsis developed Seg2RefineNet [21], a hybrid framework that combines slice-wise 2D segmentation with 3D refinement to improve spatial consistency and vessel topology preservation. In addition, Wei et al. introduced FlowVM-Net [22], which exploits optical-flow-guided temporal information fusion in XCA sequences to better preserve continuity and thin-vessel details. More recently, Ferrari et al. proposed CoroSAM [23], adapting a lightweight promptable foundation model for interactive coronary angiogram segmentation, indicating that foundation-model-based and annotation-efficient paradigms are beginning to influence coronary vessel analysis.

Despite these advancements, challenges in vessel segmentation remain, particularly in fully utilizing both high-level and low-level feature information and overcoming spatial information loss caused by convolutional layers. Recent studies indicate that future progress will likely depend not only on stronger backbones but also on better exploitation of temporal context, structural priors, annotation efficiency, and deployment efficiency. The dual U-Net++ structure proposed in this paper aims to address these issues by introducing a new model architecture that strengthens multi-resolution information interaction while avoiding explicit reliance on heavy attention mechanisms. While these various models have made significant progress in segmentation tasks, achieving an optimized solution for vessel segmentation continues to be an ongoing challenge.

### 2.2. Multi-Scale Feature Fusion Network

Multiscale feature fusion is crucial for medical image segmentation, as it can extract more useful information and improve the accuracy of semantic segmentation. Huo et al. proposed a three-branch hierarchical multiscale feature fusion network structure, HiFuse [24], which combines the advantages of Transformers and CNNs for hierarchical multiscale fusion without disrupting their respective modeling. Xie et al. proposed a feature fusion module (FFM) in FFUNet [25], which bridges the feature gap between the encoder and decoder through more efficient feature fusion with attention mechanisms and deformable convolution operations, capturing more complex correlations. Lei et al. introduced the CDFA feature aggregation module [26], which explicitly models the contrastive relationship between foreground and background features, enhancing the semantic expressiveness of the input feature maps. Previous studies have shown that multiscale feature fusion networks [27,28,29] perform very well in various image analysis tasks.

Recent work suggests that the role of multiscale fusion is evolving from simple skip-connection aggregation toward more task-aware information interaction. In SADiff [18], multiscale attention and diffusion-based refinement are coupled to suppress noise in low-contrast CTA regions while preserving tubular morphology. VM-CAGSeg [20] replaces conventional skip connections with cross-stage feature interaction fusion and introduces vessel-aware state-space blocks, demonstrating that multiscale fusion can be strengthened by explicitly incorporating geometric vessel priors and long-range dependency modeling. Seg2RefineNet [21] shows that multiscale representation learning can also be extended across dimensions, where detailed 2D slice-wise segmentation is complemented by 3D volumetric refinement to improve continuity and topology consistency. Likewise, FlowVM-Net [22] indicates that feature fusion can be expanded from spatial scales to spatiotemporal scales by integrating optical-flow-derived temporal cues into an encoder–decoder framework. Even in interactive settings, CoroSAM [23] suggests that efficient fusion between pretrained visual features and lightweight domain-specific adapters can yield accurate coronary segmentation with minimal user input.

However, these networks still face issues such as redundant skip connections, simple feature aggregation patterns, and heavy computational burdens. In particular, achieving effective interaction among shallow texture details, deep semantic context, and vascular topology information remains challenging. We therefore focus on designing a more compact and effective multi-resolution interaction mechanism that improves feature aggregation while preserving structural continuity, thereby achieving better vascular segmentation performance.

### 2.3. Thin-Vessel Segmentation and Study Positioning

Thin-vessel segmentation is particularly sensitive to foreground–background imbalance, weak local contrast, and topological discontinuity. Several studies have attempted to address this issue from different perspectives. Full-resolution designs such as FR-UNet [12] reduce information loss caused by repeated downsampling, while skeletal-prior or topology-aware methods [30] encourage the network to preserve vessel centerlines and branching structures. Integrated feature fusion models such as IMFF-Net [31] improve the use of multi-scale details in retinal vessel segmentation, and recent coronary-specific methods such as TVS-Net [16] and FlowVM-Net [22] further exploit temporal cues to enhance vascular continuity in X-ray coronary angiography. Nevertheless, many existing methods rely on additional attention modules, temporal information, structural priors, or multi-stage refinement, which may increase computational complexity or require extra data assumptions. IPSM-UNet is positioned as a convolution-based architecture that improves thin-vessel representation through multi-resolution interaction, feature aggregation, and layer-wise deep supervision without explicitly depending on heavy attention blocks.

Table 1 summarizes representative literature and clarifies the relationship between previous studies and the proposed method.

## 3. Methodology

In this section, we introduce IPSM-UNet, a convolutional neural network (CNN) architecture designed to generate precise vessel probability maps. The methodological objective is to improve the continuity and completeness of small-vessel segmentation by enhancing multi-resolution feature exchange while retaining efficient convolution-based operations.

**Figure 1:**
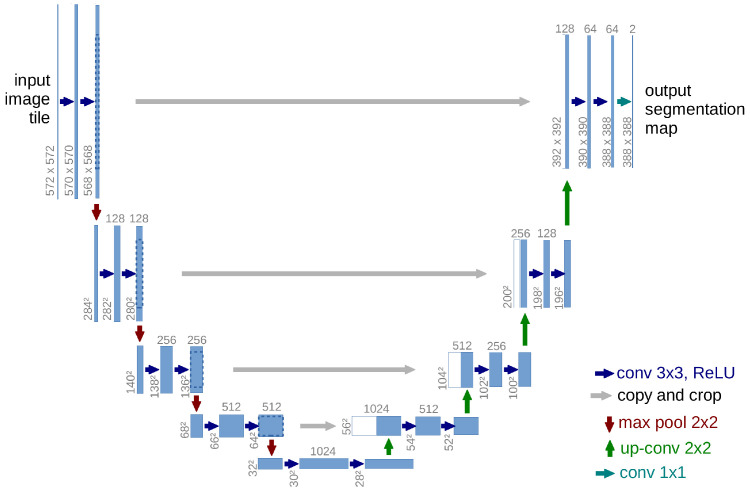
Basic U-Net architecture used as a reference. The encoder–decoder structure follows Ronneberger et al. [8], where the contracting path extracts contextual features and the expanding path restores spatial localization through skip connections.

For reference, Figure 1 illustrates the basic U-Net structure. U-Net was selected as the base family because it is a widely adopted medical image segmentation framework, its encoder–decoder design naturally combines semantic context with spatial details, and its skip connections provide a clear basis for analyzing how additional multi-resolution interaction improves vessel segmentation. Using U-Net/U-Net++ as the backbone also allows direct comparison with many vessel segmentation baselines.

The proposed algorithmic workflow is as follows. First, input images are normalized and converted into training patches. Second, IPSM-UNet extracts multi-resolution features through dual U-Net++-like branches. Third, FAMs aggregate features from adjacent sampling directions. Fourth, first-layer FAM outputs are supervised by layer-wise prediction heads. Finally, the weighted prediction map is optimized with pixel-wise BCE loss and evaluated against the full-image ground-truth mask.

### 3.1. Dual U-Net++ Feature Interaction Mechanism

**Figure 2:**
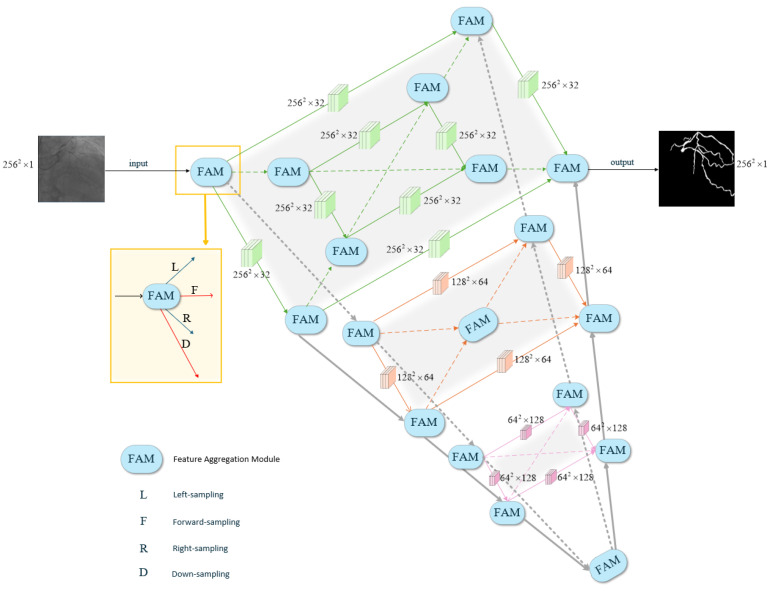
Architecture of IPSM-UNet. The network is composed of a feature aggregation module (FAM), up-sampling and down-sampling blocks, left-sampling blocks, right-sampling blocks, and forward-sampling blocks. In the first layer, deep supervision is achieved by incorporating loss functions at multiple semantic levels. In the figure, the green dashed line in the first layer, together with one downward dashed line and one upward gray solid line, forms the approximate structure and position of one U-Net++; another approximate U-Net++ structure and position is indicated by another green dashed line in the first layer, along with a downward gray dashed line and an upward gray solid line.

**Figure 3:**
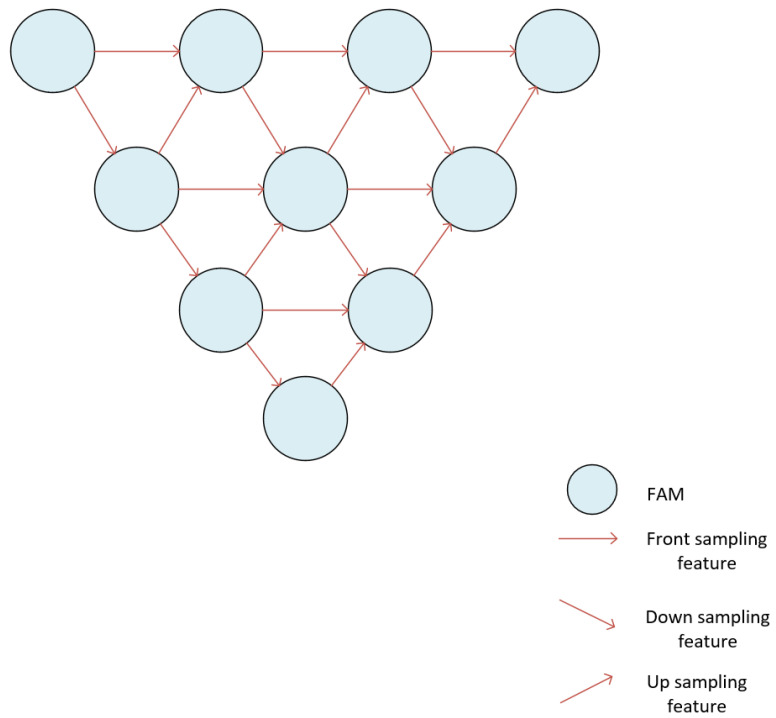
Schematic diagram of the U-Net++ architecture composed of feature aggregation modules (FAMs). The input is a feature map from the preceding sampling stage, each node denotes a FAM, and the arrows indicate forward-, down-, and up-sampling feature transfers. The output is the decoded feature map passed to the next aggregation stage or prediction head.

Figure 2 presents the architecture of IPSM-UNet. The proposed architecture comprises a three-dimensional structure formed by the intersection of two U-Net++-like frameworks, wherein interactive connections are established at each corresponding layer. To maintain clarity, certain connection lines within the U-Net++ structures are omitted in Figure 2; for detailed connection schemes, please refer to Figure 3. The diagram illustrates the interaction lines between the two U-Net++ structures, corresponding to layers 1, 2, 3, and 4 from top to bottom. In layers 2 and 4 (with layer 4 representing a Feature Aggregation Module, FAM), both layers share a central FAM for information encoding and decoding. The network executes upsampling, downsampling, left sampling, right sampling, and forward sampling, employing convolutional sampling from a three-dimensional perspective. A dual U-Net++ framework is introduced to facilitate information exchange between adjacent stages. The shallow stages provide finer semantic details, whereas the deep stages supply advanced contextual information to enhance the local receptive field of the feature maps. Consequently, the dual U-Net++ structure enables comprehensive encoding and decoding of deep feature maps by aggregating and restoring sufficient information across diverse positional contexts. Each stage aggregates feature maps from adjacent spatial dimensions during parallel expansion and learns hierarchical representations.

Furthermore, the IPSM-UNet structure achieves the concentrated and restorative effects of multiple UNet++ frameworks, resembling two UNet++ structures. Compared to FR-UNet, we expanded its multi-resolution convolution interaction mechanism while retaining the original UNet++ structure. We constructed a spatial volumetric UNet++ composed of dual UNet++ networks, significantly enhancing the diversity and effectiveness of feature aggregation without significantly increasing parameters. Given these characteristics, IPSM-UNet is suitable for dense predictions, especially for small targets with low contrast and prone to prediction errors, such as tiny blood vessels.

**Figure 4:**
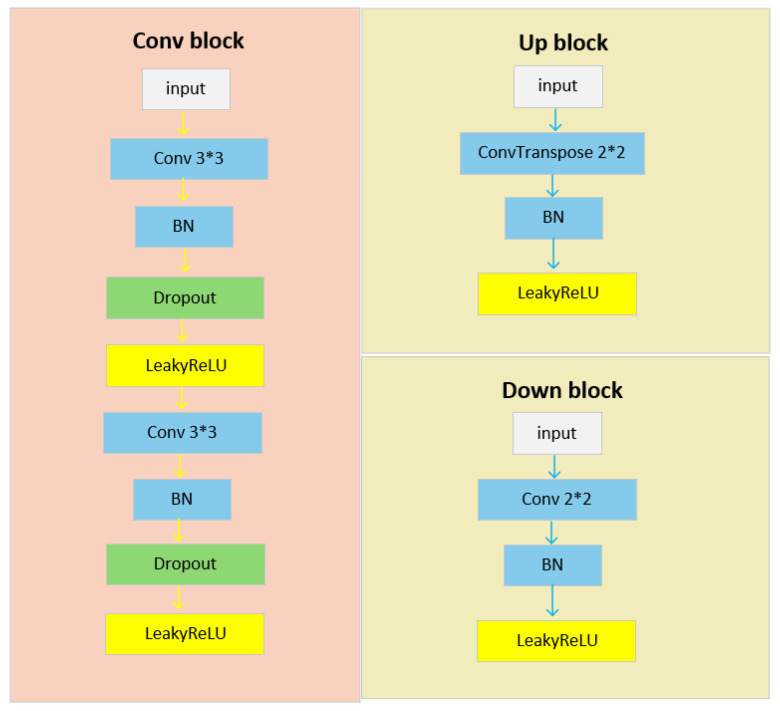
Schematic diagram illustrating the convolution block, up-sampling block, and down-sampling block. Notably, the left-sampling, right-sampling, and forward-sampling blocks are implemented as convolution blocks.

The structure of IPSM-UNet is relatively straightforward, primarily comprising a feature aggregation module, a forward-sampling stage, upsampling, downsampling, left sampling, and right sampling. As shown in Figure 4, both the upsampling and downsampling operations fundamentally employ convolutional blocks, each containing a convolution (Conv) layer, a batch normalization (BN) layer [32], and a LeakyReLU activation function with a negative slope of 0.1. Specifically, downsampling is carried out by a 2×2 convolution with a stride of 2, which reduces the spatial resolution and increases the number of feature channels, whereas upsampling is performed by a 2×2 transposed convolution with a stride of 2 to restore the spatial resolution. The network starts with 32 channels at the top level and doubles the number of channels at each subsequent level. The same convolutional operations are applied to the left sampling, right sampling, and forward-sampling stages. As illustrated by the convolution blocks in Figure 4, each of these stages contains two convolutional modules, with each module consisting of a convolution (Conv) layer, a batch normalization (BN) layer [32], a dropout layer, and a LeakyReLU activation function with a negative slope of 0.1.

### 3.2. Feature Aggregation Module

**Figure 5:**
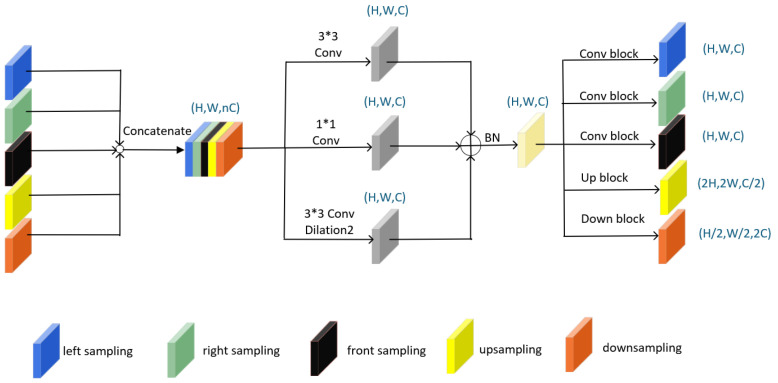
Architecture of the feature aggregation module (FAM).

The feature aggregation module (FAM) merges the feature maps from the previous residual block [33] with the up-sampling, down-sampling, left-sampling, right-sampling, and forward-sampling connections in adjacent stages. As illustrated in Figure 5, the input of an FAM is the concatenation of the available feature maps at the current node, and the output is a normalized fused feature map that can be further propagated to one or more neighboring directions. Not all FAMs take all five types of features as input, nor do they necessarily produce all five outputs; each module’s input–output pattern depends on its location within the IPSM-UNet architecture.

The three convolutional branches in Figure 5 have complementary purposes. The 1×1 convolution adjusts the channel dimension and performs local cross-channel recombination. The standard 3×3 convolution captures local vessel-edge and texture information. The dilated 3×3 convolution enlarges the receptive field without additional downsampling, which is useful for connecting discontinuous or weak vessel segments. These branches are retained together in the final FAM because they jointly encode channel-level, local, and enlarged-context information, which is complementary for thin-vessel segmentation. Removing any one of these branches would weaken one aspect of the feature representation; therefore, the final design keeps all three branches to maintain balanced channel adjustment, local boundary extraction, and contextual continuity modeling.

For example, as shown in Figure 2, in the main structure, the first FAM only receives forward-sampling features from the input image. It outputs down-sampled, left-sampled, right-sampled, and forward-sampled features but does not provide any up-sampled features. The operation is defined as

(1) $$x^{\prime }\;=\;B\left(C_{1\times 1}\left(x\right)\;+\;C_{3\times 3}\left(x\right)\;+\;C_{3\times 3,d=2}\left(x\right)\right)$$

(2) $$\mathrm{cb}\left(z\right)\;=\;\mathrm{LReLU}\left(\mathrm{Dp}\left(B(C_{3\times 3}\left(z\right))\right)\right)$$

(3) $$D\left(z\right)\;=\;\mathrm{LReLU}(B(C_{2\times 2}\left(z\right)))$$

(4) $$\begin{array}{cc} F(z)\;=\; & \mathrm{cb}\left(\mathrm{cb}(z)\right),\\ R(z)\;=\; & \mathrm{cb}\left(\mathrm{cb}(z)\right),\\ L(z)\;=\; & \mathrm{cb}\left(\mathrm{cb}(z)\right). \end{array}$$

(5) $$\begin{array}{cc} x_{D}\;=\; & D\left(x^{\prime }\right),\\ x_{F}\;=\; & F\left(x^{\prime }\right),\\ x_{R}\;=\; & R\left(x^{\prime }\right),\\ x_{L}\;=\; & L\left(x^{\prime }\right). \end{array}$$

Within these equations, B(·) denotes the batch normalization layer, C(·) represents the convolutional layer, Dp(·) corresponds to the dropout layer, and LReLU(·) indicates the leaky ReLU activation function. Similarly, F(·), R(·), L(·), and D(·) denote the forward-, right-, left-, and down-sampling operations, respectively.

Let *x* be the input image. The output $x^{\prime }$ is obtained by applying three convolutional operations, namely $C_{1\times 1}$, $C_{3\times 3}$, and $C_{3\times 3,d=2}$, to *x*; the resulting feature maps are then summed and normalized by B(·).

More concretely, the FAM begins by concatenating a selected subset of features (e.g., certain up-sampling, down-sampling, left-sampling, right-sampling, or forward-sampling features). This concatenation is then passed through two 3×3 convolutions—one of which has a dilation rate of 2—followed by a 1×1 convolution that reduces the number of channels to N×C, where *N* is the number of network layers (1, 2, 3, or 4) and *C* is the initial channel setting. Afterward, three features are summed and batch-normalized. Finally, depending on its location within the network, the FAM selectively outputs up-sampling, down-sampling, left-sampling, right-sampling, or forward-sampling features.

### 3.3. Deep Supervision

**Figure 6:**
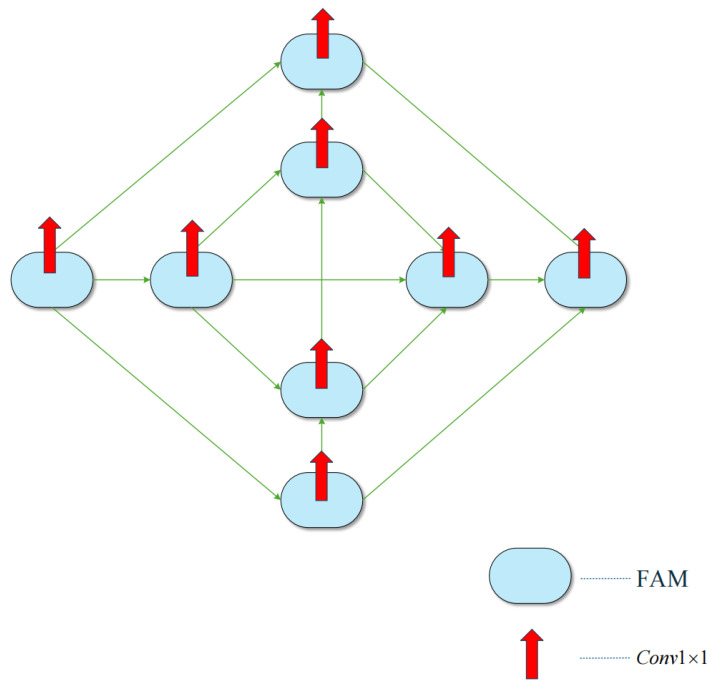
Structural diagram of the layer-wise deep supervision strategy used in the first layer of IPSM-UNet. The inputs are the forward-sampling outputs from the first-layer FAM nodes. Each red arrow denotes a 1×1 prediction head, and the outputs $y_{1},\ldots ,y_{8}$ are combined by weighted summation to form the final probability map.

In the conventional U-Net architecture, the feature maps in the shallow layers capture abundant fine-grained image details. As shown in Figure 6, to better preserve these details, we applied deep supervision to the first layer of IPSM-UNet. Specifically, a 1×1 convolution is performed on the forward-sampling output of each FAM module in this layer, yielding outputs denoted as $y_{1},y_{2},\ldots ,y_{8}$. The final prediction is obtained as the weighted sum of these outputs. The corresponding formula is as follows:

(6) $$y=\sum_{k=1}^{N}\gamma_{k}\;y_{k},$$

Here, *N* denotes the number of deeply supervised outputs and $y_{k}$ is the probability map generated by the *k*-th first-layer prediction head. The parameter $\gamma_{k}$ represents the output weight assigned to the corresponding semantic level. The weights are constrained as follows:

(7) $$\sum_{k=1}^{N}\gamma_{k}=1.$$

In this model, N=8. We use the binary cross-entropy (BCE) loss on the predicted probability map:

(8) $$L_{\mathrm{BCE}}=-\sum_{i}\left[y_{i}\log\left({\hat{y}}_{i}\right)+\left(1-y_{i}\right)\log\left(1-{\hat{y}}_{i}\right)\right],$$

where $y_{i}$ and ${\hat{y}}_{i}$ denote the ground-truth label and predicted vessel probability of pixel *i*, respectively. BCE was selected because it provides stable pixel-wise supervision for all deeply supervised outputs and allows the architectural contribution of IPSM-UNet to be evaluated under a simple and widely used objective. Dice loss and focal loss are effective alternatives for foreground–background imbalance; however, Dice-based objectives may be sensitive to very small foreground regions in patch-based training, and focal loss introduces an additional focusing parameter that requires dataset-specific tuning. We therefore used BCE as the main training objective and examined loss-function variants as a potential extension.

## 4. Experiments

### 4.1. Dataset

The DRIVE dataset [34] is widely used for retinal blood vessel segmentation and consists of 40 color retinal images with a resolution of 584 × 565 pixels, collected from a diabetic retinopathy screening program in the Netherlands. Among these images, 33 show no signs of diabetic retinopathy, while 7 exhibit mild early diabetic retinopathy. Each image is manually annotated with segmentation labels, which provide accurate references for training and evaluating segmentation models. The dataset is divided into a training set and a test set, each containing 20 images, which are used for model training and testing.

The CHASE_DB1 dataset [35] is designed for retinal vessel segmentation and contains 28 color retinal images, each with a resolution of 999 × 960 pixels, collected from the left and right eyes of 14 school children. The images are manually annotated by two independent experts, with the first annotation typically serving as the ground truth. The dataset is divided into a training set of 20 images and a test set of 8 images, which are used for model training and testing.

The DCA1 [36] dataset contains 134 X-ray coronary angiography (XCA) images with cardiologist-annotated vessel masks. The image database was provided by the Mexican Institute of Social Security. Each angiogram is a 300×300 grayscale image in portable graymap (PGM) format. Following the experimental split used in this study, 100 images were used for training, and 34 images were used for testing.

All samples in the self-collected coronary angiography image dataset were collected from the image database of the Cardiac Interventional Center of the Sixth Medical Center of the PLA General Hospital, with usage approved by the hospital’s ethics committee under public consent. The research cohort of the dataset comprises 167 patients with complex coronary lesions, with an average age of 62.99 years, a median age of 63.00 years, the youngest patient being 28 years old, and the oldest being 90 years old. This dataset includes information from 94 patients with complex coronary lesions who underwent cardiac XCA examinations between January 2019 and January 2024, yielding 595 two-dimensional images. Among these images, 500 were used as the training set and 95 as the test set.

The XCA images were acquired using three different angiography systems: Philips Medical Systems, TOSHIBA_MEC, and GE Medical Systems. They were obtained using a standardized protocol, with all patients positioned supine on the angiography table. For the left coronary artery (LCA), at least four images were acquired, including the left coronary cranial and/or left shoulder view, the left coronary spider view, the left coronary caudal and/or hepatic view, and the left coronary right shoulder view; for the right coronary artery (RCA), two images were obtained, namely the right coronary left anterior oblique view and the right coronary cranial view.

Subsequently, the XCA images were stored in Digital Imaging and Communications in Medicine (DICOM) format and further analyzed, screened, and annotated on a computer. XCA images typically include original data such as the patient’s name, ID, examination date, position angle values, and the angiography equipment. In order to protect patient privacy, the original data was removed from the DICOM files. The anonymized images and annotated images were stored in .png format, with overall dimensions of 1620 × 1620 and 1320 × 1320 pixels, and each image was approximately 1 MB in size.

For the XCA videos used to construct the dataset, images from at least six projection angles were analyzed, with each video containing approximately 21 to 163 frames. Based on the aforementioned criteria, one optimal image was selected from each viewing angle for subsequent annotation. The frame selection process emphasized three key criteria. First, images were selected based on the optimal angiographic angle within the coronary arteries to ensure effective visualization. Second, images with higher clarity and fewer vessel overlaps and motion artifacts were prioritized to maintain high image quality. Third, frames capturing the target lesions—particularly those showing stenosis and occlusion—were selected.

Adobe Photoshop (PS) software was used to annotate the XCA images, and two quality control methods were employed to ensure the high quality of the annotated images: (1) a three-level review system involving the annotating physician, a reviewing physician, and an adjudicating physician; and (2) a quality analysis based on random sampling of vascular segments, in which selectors randomly sampled certain segments according to a systematic sampling method and measurers recorded the vascular diameters of these segments in a blinded manner, comparing them with the original measurements.

**Figure 7:**
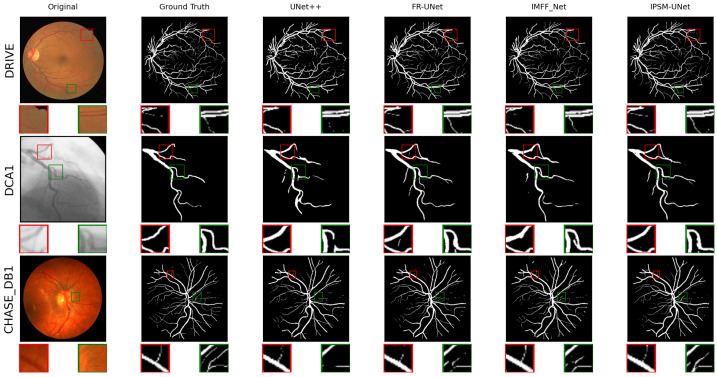
Visualization of vascular segmentation results on public datasets. From top to bottom, the images in each row are from the DRIVE, DCA1, and CHASE_DB1 datasets, respectively. From left to right, the columns show the original image, ground truth, and segmentation results from UNet++, FR-UNet, IMFF_Net, and IPSM-UNet. The DRIVE, DCA1, and CHASE_DB1 datasets contain many thin vessels; we have magnified the image details for clearer visualization, as indicated by the highlighted rectangular regions.

**Figure 8:**
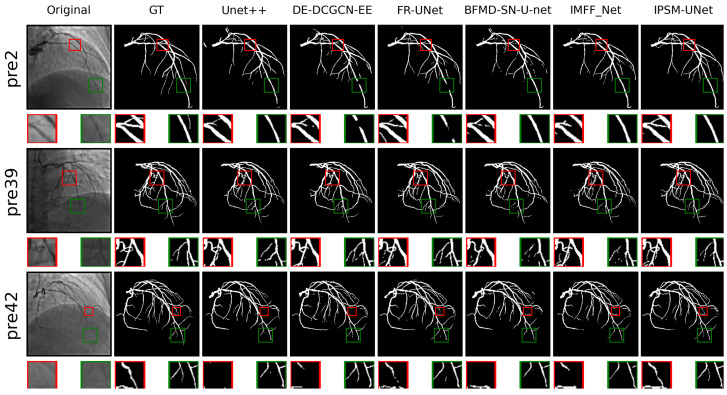
Visualization of vascular segmentation results on a private dataset. Each row shows a different test image, and columns (left to right) present the original image, ground truth, and results of representative methods, including IPSM-UNet. Red/green boxes indicate magnified regions highlighting thin-vessel continuity.

A summary of the datasets, image domains, annotation structures, and representative sample locations is provided in Table 2. Representative public-dataset samples are shown in Figure 7, and representative self-collected XCA samples are shown in Figure 8.

### 4.2. Evaluation Metrics

We evaluated segmentation performance by comparing the predicted vessel maps with the ground-truth annotations. The metrics include the area under the receiver operating characteristic curve (AUC), accuracy (Acc), sensitivity (Sen), specificity (Spe), precision (Pre), F1 score (F1), and intersection over union (IoU). Let true positives (TP) denote correctly segmented vessel pixels, true negatives (TN) denote correctly classified background pixels, false positives (FP) denote background pixels incorrectly predicted as vessels, and false negatives (FN) denote vessel pixels missed by the model. The metrics are defined as follows:

(9) $$\mathrm{Acc}=\frac{TP+TN}{TP+TN+FP+FN}$$

(10) $$\mathrm{Sen}=\frac{TP}{TP+FN}$$

(11) $$\mathrm{Spe}=\frac{TN}{TN+FP}$$

(12) $$\mathrm{Pre}=\frac{TP}{TP+FP}$$

(13) $$F1=\frac{2TP}{2TP+FP+FN}$$

(14) $$\mathrm{IoU}=\frac{TP}{TP+FP+FN}$$

In vessel segmentation, Sen measures the ability to recover true vessel regions, whereas Spe quantifies the ability to suppress background responses. Pre reflects the reliability of the predicted vessel pixels, indicating the proportion of predicted vessel pixels that are correctly classified. F1 balances missed vessels and false vessel predictions by jointly considering Sen and Pre, while IoU measures the overlap between the predicted vessel region and the ground-truth vessel region. Acc reflects the overall pixel-wise classification accuracy.

For each test image, the prediction is first obtained as a full-image vessel probability map. The probability map is then binarized using the same threshold for all methods, and TP, TN, FP, and FN are accumulated over all pixels of the entire image. Therefore, the evaluation is conducted at the pixel level over the full image rather than over bounding boxes. Patch extraction and overlapping windows are used only for training-data expansion; no bounding-box-wise measurement is involved in the reported segmentation metrics. The area under the receiver operating characteristic curve (AUC) is computed by sweeping the probability threshold over all pixels.

### 4.3. Implementation Details

We implemented IPSM-UNet using PyTorch 2.0.1 with CUDA 11.8 and performed all experiments on a single GeForce RTX 4080 Laptop GPU. Model parameters were optimized with Adam [37], using a weight decay of $1\times 10^{-5}$ and an initial learning rate of $1\times 10^{-4}$. The learning rate was scheduled by cosine annealing [38] over 40 epochs, and the model from the final epoch was used for testing. The main experimental setup is summarized in Table 3.

During training, we applied the following preprocessing and augmentation steps:

1. Color retinal images in DRIVE and CHASE_DB1 were converted into grayscale images.
2. Images from all datasets were normalized before patch extraction.
3. For DRIVE, CHASE_DB1, and DCA1, training patches were extracted using a sliding window of 48×48 with a stride of 6.
4. For the self-collected XCA dataset, training patches were extracted using a sliding window of 256×256 with a stride of 6.
5. Online data augmentation was performed on the training patches using random horizontal flipping, vertical flipping, and rotations of $90^{\circ }$, $180^{\circ }$, and $270^{\circ }$.

Patch extraction and random geometric augmentation were used only during training. During testing, each input was evaluated as a full-size image without patch extraction.

To make the data expansion process explicit, Table 4 summarizes the original image counts, train/test splits, patch settings, and the number of extracted training patches before online augmentation. The augmented counts should be calculated from the final training scripts because random flipping and rotation are applied online during training.

### 4.4. Computational Cost Analysis

To further evaluate the computational efficiency of IPSM-UNet, we compared the number of parameters, FLOPs, training time per epoch, and inference time per image with representative baseline models, including U-Net, U-Net++, and FR-UNet. All models were evaluated on the DRIVE dataset under the same experimental environment. The inference time per image denotes the average time required for a model to process one test image and generate the corresponding segmentation map during the testing stage.

As shown in Table 5, IPSM-UNet contains 17.22 M parameters and 124.86 G FLOPs. Compared with the U-Net, the proposed model introduces additional computational operations due to the dual U-Net++ feature interaction mechanism and multiple FAM modules. However, compared with the standard U-Net++ baseline, IPSM-UNet uses fewer parameters and slightly fewer FLOPs while achieving stronger vessel-segmentation performance in the main experiments. This indicates that the proposed architecture improves multi-resolution feature interaction without simply relying on a larger parameter scale.

In terms of actual running time, IPSM-UNet requires 17 min 20 s per training epoch on the DRIVE dataset and 0.0238 s per image during inference. Although this is slower than U-Net and FR-UNet, the inference time corresponds to approximately 23.8 ms per image, which remains acceptable for offline vessel segmentation and subsequent clinical image analysis. Therefore, IPSM-UNet achieves improved segmentation performance and vessel continuity with moderate additional computational overhead.

## 5. Results

### 5.1. Comparisons with SOTA Models

To highlight the superiority of the IPSM-UNet model proposed in this paper, we compared it with several state-of-the-art models from recent years using two public retinal datasets, one public coronary artery dataset, and one self-collected dataset. We evaluated the models using four metrics: sensitivity (Se), specificity (Sp), F1 score, and accuracy (Acc). Table 6 shows the results for the DRIVE dataset. The IPSM-UNet model in this paper scored 0.8310 in F1 and 0.9707 in Acc, significantly outperforming other segmentation models in terms of Acc. However, for Se and Sp, it performed slightly worse compared to the model proposed by Liu et al. [31], with a reduction of 0.0311 and 0.002, respectively. Considering all four metrics, our model has a clear advantage in Acc and F1, nearly identical performance in Sp, and a slight disadvantage in Se. For the results on the CHASE_DB1 dataset, as shown in Table 7, our model outperforms other state-of-the-art models in Se and Acc. Specifically, the Se metric is significantly better, with scores of 0.8792 and 0.9745. In terms of Sp and F1, the differences with the state-of-the-art models are 0.0057 and 0.0157, respectively. Despite slight differences in Sp and F1, our model shows a significant advantage in Se and Acc. The comparison results on the DCA1 dataset are reported in Table 8. For the DCA1 coronary artery public dataset, the proposed model outperforms other models in F1 and Acc, with scores of 0.8043 and 0.9793, respectively. The Sp score is 0.0004 lower than the state-of-the-art model, and the Se score is slightly worse. On the private dataset, the proposed model significantly outperforms other advanced models in Se, F1, and Acc, with scores of 0.8741, 0.8590, and 0.9879, respectively, while the Sp score is 0.0001 lower than the state-of-the-art model.

Based on the experimental results from the four datasets, the proposed IPSM-UNet demonstrates strong overall performance, despite minor differences in some metrics. Specifically, on the DRIVE dataset, both the Se and Sp metrics show slight reductions. On the CHASE_DB1 dataset, a slight decline in Sp and F1 scores is observed. On the DCA1 dataset, as reported in Table 8, both Se and Sp scores are slightly lower. On the private dataset, as shown in Table 9, only the Sp score shows a minor decrease. By analyzing the metrics, we can hypothesize that the model excels in overall segmentation performance and exhibits different segmentation advantages across different datasets. For example, on the CHASE_DB1 dataset, the Se metric is exceptionally high, and the F1 score shows no significant decline, indicating that the model can extract a large number of small vessels while not introducing excessive noise. For different public datasets, the excellent metrics vary. We believe this is due to the limited number of public datasets, which have not fully exploited the model’s potential; however, most metrics still surpass those of the current state-of-the-art models, demonstrating the model’s capability. When the data volume is relatively sufficient, as in the private dataset, almost all metrics are significantly better than those of the current state-of-the-art models. In summary, the IPSM-UNet network proposed in this paper has significant advantages over the state-of-the-art networks available today.

### 5.2. Cross-Dataset Validation and Domain Generalization

To further examine the robustness of IPSM-UNet against domain shift, we conducted cross-dataset validation experiments. In this setting, the model was trained on one dataset and directly evaluated on another dataset without fine-tuning. We considered two retinal-domain transfer settings, namely DRIVE-to-CHASE_DB1 and CHASE_DB1-to-DRIVE, as well as two coronary-domain transfer settings, namely DCA1-to-self-collected XCA and self-collected XCA-to-DCA1. These experiments evaluate whether the learned vessel representations can generalize to images acquired from different sources, resolutions, imaging conditions, and annotation distributions.

As shown in Table 10, IPSM-UNet maintains reasonable discriminative ability under cross-dataset evaluation, with AUC values above 0.92 in all four transfer settings. However, the segmentation-level metrics, including sensitivity, F1 score, and IoU, decrease compared with the within-dataset results, indicating that vessel segmentation remains sensitive to domain shift. In the retinal-domain transfer experiments, the CHASE_DB1-to-DRIVE setting achieves better performance than the DRIVE-to-CHASE_DB1 setting, with F1 increasing from 0.5078 to 0.6883 and IoU increasing from 0.3427 to 0.5262. This suggests that the model trained on CHASE_DB1 transfers more effectively to DRIVE, possibly because CHASE_DB1 contains higher-resolution images and more abundant small-vessel structures, which help the model learn richer retinal vessel representations. By contrast, when trained on DRIVE and tested on CHASE_DB1, the model achieves high specificity but relatively low sensitivity, indicating that it can suppress background pixels but tends to miss some vessel pixels in the target domain.

For the coronary-domain transfer experiments, the model trained on the self-collected XCA dataset and tested on DCA1 achieves an F1 score of 0.5886, a sensitivity of 0.6738, and an IoU of 0.4406. These results are better than the reverse transfer from DCA1 to the self-collected XCA dataset in terms of sensitivity, F1 score, and IoU. This asymmetric transfer performance may be attributed to the larger scale and more diverse clinical imaging conditions of the self-collected XCA dataset, which includes different angiography systems, projection views, vessel contrast patterns, and lesion-related vessel appearances. In contrast, DCA1 is smaller and has more fixed image characteristics, which may limit the diversity of learned features and reduce the model’s ability to detect low-contrast or fine coronary vessels in the self-collected clinical dataset.

Overall, the cross-dataset results indicate that IPSM-UNet has a certain degree of cross-domain robustness, especially in maintaining high AUC and specificity across different datasets. Nevertheless, the decrease in sensitivity, F1 score, and IoU under direct transfer also shows that domain shift remains an important challenge for vessel segmentation. These findings further support the need for larger multi-center datasets, domain adaptation strategies, and more robust training schemes in future work.

We further examined and visualized the vessel segmentation outcomes from UNet++, FR-UNet, IMFF_Net, and IPSM-UNet, as illustrated in Figure 7. Because retinal images contain many slender vessels, we enlarged specific rectangular areas to highlight subtle details more clearly. From the first and second sub-images in DRIVE, the two sub-images in CHASE_DB1, and the second sub-image in DCA1, one can see that IPSM-UNet detects a higher number of low-contrast, thin vessel pixels than UNet++, FR-UNet, and IMFF_Net. By contrast, as shown in the first sub-image of DCA1, IPSM-UNet exhibits fewer segmentation errors than the other techniques.

Furthermore, IPSM-UNet achieved impressive performance on coronary angiography images from a private dataset. We randomly selected three test images—the 2nd, 39th, and 42nd—and denoted them as pre2, pre39, and pre42, as illustrated in Figure 8. In pre2, the area highlighted by the red box demonstrates that IPSM-UNet yields improved connectivity, resulting in more continuous vessel segmentation. In pre42, the region marked in green shows that IPSM-UNet can delineate vessels with greater precision. By preserving more spatial information of the vessels for interactive fusion, enhancing the encoder’s ability to integrate information, and bolstering the decoder’s capacity to retain details, IPSM-UNet produces outputs that more closely approximate the ground-truth annotations compared to other methods. Overall, this comparison underscores the robust capability of IPSM-UNet in vessel segmentation.

### 5.3. Error Analysis and Failure Cases

Although IPSM-UNet improves overall segmentation quality, it does not outperform competing methods in every local region. Table 11 reports a quantitative error summary derived from the proposed method’s sensitivity and specificity. The false-negative rate (FNR) is calculated as 1−Sen and reflects missed vessel pixels, while the false-positive rate (FPR) is calculated as 1−Spe and reflects background pixels incorrectly classified as vessels.

The main failure cases are as follows. First, extremely low-contrast distal vessels may still be missed, leading to local false negatives and lower Sen, especially in datasets with weak vessel boundaries, such as DCA1. Second, noisy background structures, vessel-like shadows, or overlapping anatomical structures may occasionally be classified as vessel pixels, leading to false positives. Third, when a vessel segment is very close to the background intensity or overlaps with other structures, IPSM-UNet may perform similarly to or slightly worse than competing methods in that local region. These observations suggest that the proposed feature interaction improves average continuity but cannot completely eliminate the ambiguity caused by severe low contrast and imaging noise.

### 5.4. Ablation Studies

As shown in Figure 2, IPSM-UNet can be regarded as a spatial U-Net in which the main U-Net++ architecture and the auxiliary U-Net++ architecture partially overlap. Thus, IPSM-UNet can be considered as an encoder- = decoder architecture composed of the U-Net++ baseline, the dual U-Net++ feature interaction mechanism (DUFIM), the Feature Aggregation Module (FAM), and Layer-wise deep supervision (LWDS). To validate the effectiveness of these modules, we conducted ablation experiments on the DRIVE dataset. In these experiments, we used U-Net++ as the baseline and incrementally added different components while keeping all hyperparameters identical. As shown in Table 12, we observe that the dual UNet++ feature interaction mechanism (referred to as “baseline + DUFIM”) significantly improves overall performance compared to UNet++. The Se, Sp, F1, and Acc values increase by 0.0285, 0.0008, 0.0186, and 0.0029, respectively. These experimental results demonstrate that the dual UNet++ feature interaction mechanism enhances vascular segmentation performance. By combining UNet++ with the dual UNet++ feature interaction mechanism, we form the core of IPSM-UNet.

We then add a feature aggregation module (FAM) and layer-wise deep supervision (LWDS) to this architecture. The experimental results are reported in Table 12 as “baseline + DUFIM + FAM,” “baseline + DUFIM + LWDS,” and “baseline + DUFIM + FAM + LWDS.” The feature aggregation module uses multiple parallel convolutions. While other metrics remain approximately the same, it significantly improves the Se score. Notably, “baseline + DUFIM + FAM” outperforms “baseline + DUFIM,” with Se increasing by 0.38%. We believe this method gathers richer representational information, which helps segment vascular pixels characterized by extremely imbalanced classes and increases the likelihood of capturing tiny vessels.

Deep supervision, as a practical module, has been widely used in many popular network architectures. Here, we apply it to supervise the dual U-Net++ feature interaction mechanism feature maps. Compared with “baseline + DUFIM,” deep supervision increases the Sp and Acc scores but reduces Se. This indicates that deep supervision constrains the model’s prediction of small vessels, thus avoiding excessive classification of noise and shadows as vessels. Finally, we further embed both FAM and LWDS into the baseline (referred to as “baseline + DUFIM + FAM + LWDS”) to verify the combined effects of all components. As shown in Table 12, this method achieves the highest Sen and F1 scores in the ablation study, with only minimal differences in other metrics, demonstrating the effectiveness of IPSM-UNet in vascular segmentation.

In summary, IPSM-UNet retains full-resolution representation learning, integrating deep supervision and the feature aggregation module, and can be applied to vascular segmentation.

## 6. Discussion

The results support the initial hypothesis that strengthening multi-resolution interaction and shallow-layer supervision can improve vessel segmentation, especially for thin and low-contrast structures. Compared with conventional U-Net-like architectures, IPSM-UNet introduces a dual U-Net++-like inverted pyramid structure to increase feature exchange across resolutions. The FAM further aggregates channel-adjusted, local, and dilated contextual features, and layer-wise deep supervision encourages first-layer FAM nodes to contribute directly to the final prediction.

### Limitations and Future Work

This study has several limitations. First, although IPSM-UNet is evaluated on retinal and coronary datasets, domain shift remains challenging because different datasets vary in imaging modality, resolution, contrast, annotation protocol, and vessel morphology. Cross-dataset validation in Table 10 shows that IPSM-UNet maintains high AUC and specificity under direct transfer, but sensitivity, F1 score, and IoU still decrease across domains, indicating that domain shift remains challenging. Second, the current error analysis shows that extremely low-contrast distal vessels and noisy vessel-like background regions remain difficult. Future work may incorporate topology-aware losses, uncertainty estimation, or post-processing strategies to further reduce false negatives and false positives. Third, the present model uses BCE loss as the main objective; Dice loss, focal loss, or hybrid objectives may be explored in future experiments to better handle class imbalance. Fourth, although the proposed method avoids heavy attention mechanisms, the dual U-Net++-like design and multiple FAMs still introduce additional convolutional cost. As reported in Table 5, IPSM-UNet requires higher FLOPs and longer training time than simpler U-Net-based models, although its inference time remains acceptable for offline vessel segmentation. Finally, the internal dataset is retrospective and collected from a single medical center; larger, multi-center external validation would further strengthen the clinical generalizability of the method.

## 7. Conclusions

This paper proposes IPSM-UNet, an inverted pyramid-shaped dual U-Net++ architecture for coronary artery segmentation. The method combines multi-resolution feature interaction, FAM-based aggregation, and layer-wise deep supervision to improve the continuity and completeness of vessel segmentation. Experiments on DRIVE, CHASE_DB1, DCA1, and a self-collected XCA dataset demonstrate competitive or superior performance across multiple metrics. Qualitative visualization and error analysis further indicate that IPSM-UNet is effective for small-vessel extraction but may still be affected by extremely low contrast, noise, and domain shift. Future work will focus on cross-domain robustness, topology-preserving objectives, loss-function variants, and more efficient architecture design.

## Figures and Tables

**Table 1:** Summary of representative vessel segmentation methods and their limitations.

Category	Representative Idea and Limitation	Relation to This Study
U-Net-based methods	Encoder–decoder networks with skip connections are widely used for medical image segmentation, but they may be insufficient for recovering very thin and low-contrast vessels.	U-Net provides the basic segmentation framework for this study.
U-Net++ and dense-skip methods	Nested or dense-skip connections improve multi-scale feature reuse, but redundant pathways may still limit effective cross-resolution interaction.	This motivates the dual U-Net++ feature interaction mechanism in IPSM-UNet.
Attention-based methods	Spatial or channel attention can enhance global dependency modeling, but heavy attention modules often increase computational cost and training burden.	IPSM-UNet aims to improve feature interaction without relying on heavy attention mechanisms.
Multi-scale fusion methods	Multi-scale fusion combines features from different resolutions, but existing fusion strategies may be local or insufficient for maintaining thin-vessel continuity.	The proposed FAM strengthens structured multi-resolution aggregation for vessel segmentation.

**Table 2:** Summary of datasets used in this study. The sample column indicates where representative images of each dataset are visualized in the manuscript.

Dataset	Source/Domain	Images and Split	Annotation	Main Characteristics	Sample
DRIVE	Public retinal fundus images	40 images; 20/20 train/test	Binary vessel masks	Color fundus images containing many thin retinal vessels; images were converted to grayscale before training.	Figure 7, DRIVE row
CHASE_DB1	Public retinal fundus images	28 images; 20/8 train/test	Binary vessel masks	High-resolution fundus images from both eyes, with abundant small-vessel structures and variable vessel contrast.	Figure 7, CHASE_DB1 row
DCA1	Public XCA images	134 images; 100/34 train/test	Cardiologist-labeled coronary vessel masks	Grayscale X-ray coronary angiography images with low vessel contrast and more background interference than retinal images.	Figure 7, DCA1 row
Self-collected XCA	Sixth Medical Center of PLA General Hospital	595 images; 500/95 train/test	Expert-reviewed coronary vessel masks	Multi-device XCA data from patients with complex coronary lesions; all images were anonymized and stored in PNG format.	Figure 8

**Table 3:** Main experimental configuration used in this study.

Item	Setting
Framework	PyTorch
GPU	GeForce RTX 4080 Laptop GPU
Optimizer	Adam
Initial learning rate	$1\times 10^{-4}$
Weight decay	$1\times 10^{-5}$
Learning-rate schedule	Cosine annealing
Epochs	40
Training augmentation	Horizontal flip, vertical flip, and $90^{\circ }$/$180^{\circ }$/$270^{\circ }$ rotations
Testing strategy	Full-image inference and pixel-wise evaluation

**Table 4:** Dataset split and training patch extraction settings. The reported training-patch numbers denote the extracted patches before online random augmentation.

Dataset	Original Images	Train/Test	Patch Size	Stride	Training Patches Before Online Augmentation
DRIVE	40	20/20	48×48	6	160,160
CHASE_DB1	28	20/8	48×48	6	492,800
DCA1	134	100/34	48×48	6	193,600
Self-collected XCA	595	500/95	256×256	6	40,500

**Table 5:** Computational cost comparison of different segmentation models on the DRIVE dataset. Params and FLOPs were calculated under the same input setting. Training time and inference time were measured on the same hardware platform.

Method	Params (M)	FLOPs (G)	Training Time/Epoch	Inference Time/Image
U-Net	13.39	30.93	8 min 20 s	0.0123 s
U-Net++	24.82	133.48	13 min 33 s	0.0224 s
FR-UNet	5.72	58.90	15 min 12 s	0.0212 s
IPSM-UNet	17.22	124.86	17 min 20 s	0.0238 s

**Table 6:** Comparison results of different segmentation methods on the DRIVE dataset.

Method	Year	Se	Sp	F1	Acc
Sule et al. [39]	2020	0.7092	0.9820	-	0.9447
Khan et al. [40]	2020	0.7970	0.9730	-	0.9580
Chen et al. [41]	2021	0.7858	0.9832	0.8214	0.9550
Tan et al. [30]	2022	0.8323	0.9859	-	0.9461
Liu et al. [12]	2022	0.8248	0.9848	0.8303	0.9706
Zhong et al. [42]	2022	0.8074	0.9755	-	0.9607
Li et al. [43]	2022	0.8138	0.9763	0.8236	0.9556
Dong et al. [44]	2022	0.7954	-	-	0.9586
Liu et al. [45]	2023	0.8164	0.9764	0.8254	0.9591
Wei et al. [46]	2023	0.8018	0.9826	0.8302	0.9581
Gao et al. [47]	2023	0.8315	0.9766	0.8301	0.9586
Liu et al. [31]	2024	**0.8575**	**0.9860**	0.7977	0.9621
IPSM-UNet (Proposed)	-	0.8264	0.9848	**0.8310**	**0.9707**

Note: Bold values indicate the best performance for each metric.

**Table 7:** Comparison results of different segmentation methods on the CHASE_DB1 dataset.

Method	Year	Se	Sp	F1	Acc
Guo et al. [48]	2019	0.7888	0.9801	0.7983	0.9627
Wang et al. [49]	2020	0.7948	0.9842	0.8220	0.9648
Khan et al. [50]	2020	0.8440	0.9810	-	0.9722
Wang et al. [51]	2020	0.8427	0.9836	0.8105	0.9706
Yang et al. [52]	2021	0.8176	0.9776	0.7997	0.9667
Tan et al. [30]	2022	0.7817	0.9794	-	0.9561
Zhong et al. [42]	2022	0.8440	0.9728	-	0.9517
Li et al. [43]	2022	0.8053	0.9835	0.8076	0.9575
Liu et al. [12]	2022	0.8767	0.9816	0.8141	**0.9748**
Wei et al. [46]	2023	0.8138	0.9824	0.8196	0.9678
Liu et al. [45]	2023	0.8284	0.9821	**0.8349**	0.9664
Liu et al. [31]	2024	0.8048	**0.9867**	0.7894	0.9730
IPSM-UNet (Proposed)	-	**0.8792**	0.9810	0.8129	0.9745

Note: Bold values indicate the best performance for each metric.

**Table 8:** Comparison results of different segmentation methods on the DCA1 dataset.

Method	Year	Se	Sp	F1	Acc
Ronneberger et al. [8]	2015	0.7816	0.9866	0.7735	0.9758
Zhou et al. [10]	2018	0.7954	0.9862	0.7786	0.9761
Oktay, Ozan, et al. [53]	2018	0.7986	0.9853	0.7748	0.9755
Sun, Ke, et al. [54]	2019	0.8007	0.9876	0.7919	0.9777
Mou, Lei, et al. [55]	2019	0.7895	0.9867	0.7790	0.9763
Liu et al. [12]	2022	0.8248	0.9875	0.8022	0.9788
Kus, Zeki. [56]	2023	**0.8412**	0.9889	0.7822	**0.9793**
Zhang et al. [57]	2024	0.7779	**0.9891**	0.7830	0.9777
Asif, Sohaib, et al. [58]	2024	0.8237	-	0.8029	0.9778
Liu et al. [31]	2024	0.8072	0.9874	0.7909	0.9778
IPSM-UNet (Proposed)	-	0.8134	0.9887	**0.8043**	**0.9793**

Note: Bold values indicate the best performance for each metric.

**Table 9:** Comparison results of different segmentation methods on the self-collected dataset.

Method	Year	Se	Sp	F1	Acc
Ronneberger et al. [8]	2015	0.8234	0.9913	0.8170	0.9842
Zhou et al. [10]	2018	0.8267	0.9910	0.8167	0.9840
Liu et al. [12]	2022	0.8199	**0.9929**	0.8286	0.9856
Li, Yang, et al. [59]	2022	0.8564	0.9912	0.8341	0.9855
Deari et al. [60]	2023	0.8034	0.9912	0.8026	0.9833
Tan et al. [61]	2024	0.8619	0.9914	0.8402	0.9859
Liu et al. [31]	2024	0.8513	0.9925	0.8445	0.9865
IPSM-UNet (Proposed)	-	**0.8741**	0.9928	**0.8590**	**0.9879**

Note: Bold values indicate the best performance for each metric.

**Table 10:** Cross-dataset validation results for evaluating domain generalization. The model was trained on the source dataset and directly tested on the target dataset without fine-tuning.

Training Dataset	Testing Dataset	AUC	F1	Acc	Sen	Spe	Pre	IoU
DRIVE	CHASE_DB1	0.9213	0.5078	0.9506	0.4117	0.9869	0.6777	0.3427
CHASE_DB1	DRIVE	0.9571	0.6883	0.9560	0.5583	0.9944	0.9062	0.5262
DCA1	Self-collected XCA	0.9438	0.4863	0.9651	0.3860	0.9921	0.6767	0.3301
Self-collected XCA	DCA1	0.9222	0.5886	0.9587	0.6738	0.9752	0.5723	0.4406

**Table 11:** Pixel-level error analysis of IPSM-UNet based on the reported sensitivity and specificity.

Dataset	Sen	Spe	FNR	FPR
DRIVE	0.8264	0.9848	0.1736	0.0152
CHASE_DB1	0.8792	0.9810	0.1208	0.0190
DCA1	0.8134	0.9887	0.1866	0.0113
Self-collected XCA	0.8741	0.9928	0.1259	0.0072

**Table 12:** Ablation study of IPSM-UNet with different network configurations in vessel segmentation.

Method	Se	Sp	F1	Acc
baseline	0.7891	0.9850	0.8114	0.9679
baseline + DUFIM	0.8176	0.9858	0.8300	0.9708
baseline + DUFIM + FAM	0.8214	0.9853	0.8304	0.9708
baseline + DUFIM + LWDS	0.8072	**0.9870**	0.8292	**0.9710**
baseline + DUFIM + FAM + LWDS	**0.8264**	0.9848	**0.8310**	0.9707

Note: Bold values indicate the best performance for each metric.

## Data Availability

The public datasets analyzed in this study are available from their respective official repositories. The internal coronary angiography dataset is available from the corresponding author upon reasonable request and with permission from the relevant institution. The internal data are not publicly available due to privacy and ethical restrictions related to patient medical images.

## Abbreviations

The following abbreviations are used in this manuscript:

IPSM	Inverted Pyramid-Shaped Multi-resolution
XCA	X-ray coronary angiography
FAM	Feature aggregation module
DUFIM	Dual U-Net++ feature interaction mechanism
LWDS	Layer-wise deep supervision
CNN	Convolutional neural network
FCN	Fully convolutional network
BN	Batch normalization
BCE	Binary cross-entropy
AUC	Area under the receiver operating characteristic curve
Acc	Accuracy
Sen	Sensitivity
Spe	Specificity
Pre	Precision
IoU	Intersection over union
TP	True positive
TN	True negative
FP	False positive
FN	False negative
FNR	False-negative rate
FPR	False-positive rate
DICOM	Digital Imaging and Communications in Medicine
LCA	Left coronary artery
RCA	Right coronary artery
